# Lactate clearance as a simple bedside instrument to predict short-term mortality of severe septic patients

**DOI:** 10.1186/cc10412

**Published:** 2011-10-27

**Authors:** W Hambali, K Chen, D Widodo, E Dewiasty, HT Pohan, S Suwarto

**Affiliations:** 1 Internal Medicine Department, Faculty of Medicine, University of Indonesia, Jakarta, Indonesia; 2 The Indonesian Society for the Study of Tropical Medicine and Infectious Diseases, Jakarta, Indonesia

## Introduction

Severe sepsis is major health problem with a high mortality rate, and still its incidence continues to rise [1-5]. Lactate clearance, measurement of the lactate level at two consecutive times, is an inexpensive and simple clinical parameter that can be obtained by a minimally invasive means [6-8]. This parameter represents kinetic alteration of the anaerobic metabolism that makes it a potential parameter to evaluate disease severity and intervention adequacy. Lactate clearance early in the hospital course may indicate a resolution of global tissue hypoxia and is associated with improved outcome [7-9]. Nevertheless, the relationship between lactate clearance and short-term mortality in severe septic patients is still poorly understood. Understanding the presence of confounder factors is also important to strengthen the role of lactate clearance in the treatment of severe septic patients.

## Objective

To evaluate the clinical course between lactate clearance groups, and determine the role of confounder variables that influence its relationship.

## Methods

This is a prospective cohort study conducted in Ciptomangun-kusumo Hospital, from March to May 2011. Patients were categorized into the high lactate clearance group if there were differences in 6-hour lactate levels ≥10%, and conversely were categorized into the low lactate clearance group [7,8]. Deaths were observed within the first 10 days. After data collection, the statistical methods were analyzed using survival analysis. Analysis of confounder variables was performed by multivariate Cox regression test.

## Results

**Figure 1:**
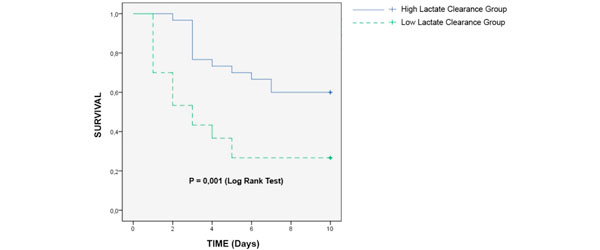
**Kaplan-Meier curves between lactate clearance groups**.

During the research period there were 60 patients recruited, consisting of 30 patients grouped into high lactate clearance and the remainder grouped into low lactate clearance. The survival rates in high and low lactate clearance groups were 60.0% versus 26.7% (see Figure 1). In the low lactate clearance group the median survival was 3 days, while the mortality rate did not reach 50% in the high lactate clearance group. The first interquartile was 1 day and 4 days. The hazard ratio between groups was 2.87 (95% CI = 1.41 to 5.83). Steps taken to analyze the role of variables that potentially act as confounder factors were by using bivariate analysis, in which variables that influenced the occurrence of deaths (indicated by *P *< 0.25) underwent multivariate analysis subsequently. On multivariate analysis the presence of septic shock, degree of organ dysfunction, vasoactive drug usage, blood transfusion, and fluid resuscitation change the hazard ratio by no more than 10% (Table 1). For that reason, these parameters were not considered as confounders.

**Table 1:** Variables that potentially act as confounder factors

Variable	Nonsurvivors	Survivors	*P *value	Adjusted HR (95% CI)	HR change (%)
Septic shock within 6 hours					
With septic shock	11	4	0.081	3.083 (1.505 to 6.317)	7.4
Without septic shock	23	22			
Initial SOFA score					
>9	11	3	0.038	2.919 (1.388 to 6.138)	1.7
≤9	19	21			
Vasoactive drugs within 6 hours					
Without vasoactive drugs	22	23	0.013	2.988 (1.462 to 6.106)	4.1
With vasoactive drugs	12	3			
Invasive ventilation within 6 hours^a^					
Without mechanical ventilation	31	23	0.777	-	-
With mechanical ventilation	3	3			
PRC transfusion within 6 hours					
Without transfusion	29	25	0.069	3.077 (1.493 to 6.340)	7.2
With transfusion	5	1			
Fluid resuscitation within 6 hours					
<1,000 cm^3^	15	18	0.166	2.942 (1.444 to 5.994)	2.5
≥1,000 cm^3^	19	8			

HR, hazard ratio; SOFA, Sequential Organ Failure Assessment; PRC, packed red cells. ^a^Invasive ventilation parameter not included in multivariate analysis, because *P *< 0.25.

## Conclusion

Severe septic patients with high lactate clearance have a better survival rate compared with the low lactate clearance group, and its relationship is not influenced by the presence of confounder variables.
